# Effect of Ultrasound-Assisted Solvent Enzymatic Extraction on Fatty Acid Profiles, Physicochemical Properties, Bioactive Compounds, and Antioxidant Activity of *Elaeagnus mollis* Oil

**DOI:** 10.3390/foods11030359

**Published:** 2022-01-26

**Authors:** Xiaorui Lu, Hongmiao Du, Yuanyuan Liu, Yong Wang, Dong Li, Lijun Wang

**Affiliations:** 1 Beijing Advanced Innovation Center for Food Nutrition and Human Health, National Energy R & D Center for Non-Food Biomass, College of Engineering, China Agricultural University, P.O. Box 50, 17 Qinghua Donglu, Beijing 100083, China; luxiaorui118@163.com (X.L.); liu-yy@cau.edu.cn (Y.L.); 2 Beijing Products Quality Supervision and Inspection Institute, 9 Shunxing Road, Beijing 101300, China; duhongmiao@126.com; 3 School of Chemical Engineering, UNSW, Sydney, NSW 2052, Australia; Yong.wang2@unsw.edu.au; 4 Beijing Key Laboratory of Functional Food from Plant Resources, College of Food Science and Nutritional Engineering, China Agricultural University, 17 Qinghua Donglu, Beijing 100083, China

**Keywords:** ultrasound-assisted solvent enzymatic extraction (UASEE), *Elaeagnus mollis* oil, physicochemical properties, antioxidant activity, thermal behavior

## Abstract

*Elaeagnus mollis* oil extracted from the nuts of *Elaeagnus mollis* Diels can be used in food and pharmaceutical applications due to its excellent nutritional value. An ultrasound-assisted solvent enzymatic extraction (UASEE) method was used to extract oil from *Elaeagnus mollis* Diels with *n*-hexane solvent (1:11.6 g/mL) and 1.1% (*w*/*w*) mixed enzymes (neutral protease:hemicellulase:pectinase = 1:1:1, *w*/*w*/*w*). The physicochemical properties, fatty acid profile, bioactive compounds, antioxidant activity, morphology, and thermal stability of UASEE oil were investigated and compared with soxhlet extraction (SE) oil and cold pressing (CP) oil. The UASEE oil exhibited a higher content of unsaturated fatty acids (93.96 ± 0.28%), total tocopherols and tocotrienols (147.32 ± 2.19 mg/100 g), total phytosterols (261.78 ± 5.74 mg/100 g), squalene (96.75 ± 0.31 mg/100 g), total phenolic content (84.76 ± 2.37 mg GAE/kg), and antioxidant activity (12.52 ± 0.28 mg/mL) than SE and CP oil. The lower peroxide value and acid value in UASEE oil indicated its better quality and lower likelihood of rancidity. The oil obtained using UASEE had higher thermal stability as well, as indicated by thermogravimetric analysis. Scanning electron microscopy (SEM) showed that the UASEE process causes damage to cell walls, and the leakage of substances in the cells facilitates extraction in the following step. Thus, UASEE is a promising processing method for the extraction of *Elaeagnus mollis* oil.

## 1. Introduction

Currently, health-conscious consumers drive demand for safer and higher-quality products in the global marketplace. In particular, oil extracted from plant species which have medical and health functions is gaining increased attention [1]. Many studies have investigated biological properties of plant oils including their antimicrobial, antifungal, insecticidal, antioxidant, and anticancer uses [2,3,4,5]. The extracted plant-based oils are generally used in cooking, cosmetics, health supplement capsules, and other purposes [6].

*Elaeagnus mollis* (*E. mollis*) oil is a new commercial product with excellent nutritional value. The oleic and linoleic acid contents in *E. mollis* oil are over 80%. In addition, *E. mollis* oil is described as being rich in tocopherols, tocotrienols, squalene, phytosterol, and phenolic content [7,8]. *E. mollis* oil is considered a nutritional food and has attracted the interest of health-conscious consumers because of its pharmacological activities [9,10,11].

Cold pressing (CP) and solvent extraction (SE) are two conventional and common methods for the extraction of *E. mollis* oil. CP is able to better preserve the native attributes of oil such as flavor and color, although low oil yields limit its industrial application [12]. SE is prevalently used in the food and pharmaceutical industries for its high oil extraction efficiency; however, the high extraction temperature and long extraction time required lead to a loss of bioactive phytochemicals [13]. Therefore, it is essential to explore an effective extraction method for oil in order to improve the efficiency of extraction and preserve the quality attributes of the oil.

Compared with the two conventional extraction technologies, the UASEE process of enzymatic extraction is stimulated by ultrasonic waves which break the cell walls to permit enzyme-aided reactions and the consequent discharge of targeted components [14]. Heidari and Dinani [15] found that using an ultrasonic process combined with enzymatic treatment obtained a higher yield of peanut oil. Ghasemi and Dinani [6] reported this combined treatment could obtain higher oil yields and enhance the release of total phenolic content by disrupting walnut cells. Similarly, Amigh and Dinani [16] applied the combined treatment for promoting oil content in date seeds.

Recent studies of *E. mollis* oil have focused on optimal extraction conditions [8,17]. Lu, et al. [18] illustrated that ultrasound-assisted enzymatic extraction using an *n*-hexane solvent method for *E. mollis* oil extraction has the advantages of high oil yield and lower consumption of both energy and time. However, there have been no comprehensive studies comparing the quality of *E. mollis* oil obtained using UASEE and traditional methods. In addition, the comparison of changes to the microstructure of *E. mollis* seeds between before and after extraction by UASEE has not been previously reported.

The current research study evaluates the fatty acid profile, physicochemical properties, bioactive compounds (tocopherols, tocotrienols, squalene, phytosterol, and phenolic contents), antioxidant activity, and thermal behavior of UASEE oil and compares it with oil obtained using the SE and CP methods. In addition, we observe the differences in the microstructure of *E. mollis* before and after extraction using the UASEE process.

## 2. Materials and Methods

### 2.1. Chemicals and Plant Materials

Fatty acid methyl ester, tocopherols (α, β, γ, and δ), tocotrienols (α, β, γ, and δ), phytosterol, squalene, 4-hydroxybenzoic acid, caffeic acid, chlorogenic acid, cinnamic acid, gallic acid, vanillic acid, p-coumaric acid, sinapic acid, ferulic acid, ellagic acid, salicylic acid, vanillin, epicatechin, quercetin and 2,2-diphenyl-1-picrylhydrazyl (DPPH) were obtained from Sigma-Aldrich Chemical Co., Ltd. (Shanghai, China). Folin-Ciocalteu reagent, chromatographic grade methanol, acetonitrile, formic acid and *n*-hexane were purchased from Thermo Scientific (Beijing, China). All reagents and chemicals were kept at analytical grade and were purchased from the Sinopharm Chemical Reagent Beijing Co., Ltd., (Beijing, China).

The *E. mollis* seeds used in this study were bought from a local market in Yi cheng, Shanxi province, China. The collected seeds were manually de-hulled and air-dried until the moisture content was below 5%. Then, the kernels were ground with a disintegrator and passed through a 40-mesh sieve to obtain a homogeneous and fine powder, which was stored in polyethylene containers at 4 °C until analysis.

### 2.2. Oil Extraction

#### 2.2.1. UASEE

*E. mollis* seed oil extraction was carried out according as in our previous paper [18]. The powder was mixed with *n*-hexane (1:11.6 g/mL) at room temperature, followed by ultrasonication at a power of 583 W using an ultrasonic bath (KH-250DE, Kunshan, China) at 40 °C for 60 min. Mixed enzymes of 1.1% *w*/*w* (neutral protease/hemicellulase/pectinase in equal proportions) were adjusted to pH 5.0 by aqueous sodium hydrogen phosphate (0.2 M) and aqueous citric acid (0.1 M) buffer solution and added to the ultrasonic extracted solutions. Then, the mixtures were incubated in a shaker at a rate of 100 rpm for 2 h at 50 °C. The supernatant was filtered and the solvent was removed by a rotary vacuum evaporator (RV 8, IKA, Staufen, Germany) at 50 °C.

#### 2.2.2. SE

A slightly modified soxhlet standard extraction method as described by the Association of Official Analytical Chemists (AOAC) [19] was used. The powder (10 g) was mixed with *n*-hexane (100 mL) using a soxhlet extractor at 80 °C for 9 h, followed by *n*-hexane separation using a rotary vacuum evaporator at 50 °C.

#### 2.2.3. CP

*E. mollis* oil was extracted by cold pressing for comparison using a mechanical screw press (ZJ-420, Dongguan, Guangdong, China).

The collected oil was centrifuged at 4 °C for 5 min at 9500 rpm and stored at 4 °C in brown bottles for further analysis.

### 2.3. Physicochemical Characteristics

The specific gravity, refractive index, saponification value, iodine value, peroxide value, and acid value were determined according to the AOAC official methods [19].

### 2.4. Fatty Acid Profiles

Fatty acid profiles were determined using a chromatograph–mass spectrometer (7890N-5795C, Agilent Technologies, CA, USA), equipped with a CP-Sil 88 capillary column (100 m × 0.25 mm, 0.20 µm film thickness, Agilent Technologies, CA, USA). Prior to analysis, the oil was transformed into fatty acid methyl esters (FAMEs) according to the method reported by Naik, et al. [20]. Both the injector and the detector temperatures were maintained at 250 °C. The oven temperature was programmed isothermally to 140 °C for 5 min and then heated to 210 °C (4 °C/min) for 5 min. The injected volume was 1.0 µL and the split rate was 1:20. The fatty acid profiles were identified according to their chromatographic and mass spectral characteristics (GC-MS databases, NIST.11) and retention time.

### 2.5. Bioactive Compounds

#### 2.5.1. Tocopherols and Tocotrienols

For quantification of tocopherols and tocotrienols, the oil was saponified, then the compounds were determined by the HPLC method as described by Ben Mohamed et al. [21] with minor changes. The contents of tocopherols and tocotrienols were measured by an LC-20A high-performance liquid chromatography (Shimadzu, Kyoto, Japan) using a C30 column (4.6 mm × 250 mm, 5 µm, Shimadzu, Kyoto, Japan) and an RF-20A xs fluorescence detector (Shimadzu, Kyoto, Japan).

#### 2.5.2. Squalene

For analysis of squalene, oil samples were prepared based on the official method NY/T 3673-2020 of the Ministry of Agriculture of China, with slight modifications [22]. using a chromatograph-mass spectrometer (GC-QP2010, Shimadzu, Kyoto, Japan) equipped with an RTX-5MS capillary column (30 m × 0.25 mm, 0.25 µm film thickness, Shimadzu, Kyoto, Japan). The column temperature was set at 200 °C and held for 1 min; then, the temperature was raised at a rate of 25 °C/min and maintained at 300 °C for 5 min. The source temperature was 230 °C, the split rate was 1/50, and the injection volume was 1.0 µL. Squalene was identified and calculated using the external standard to create a calibration curve.

#### 2.5.3. Phytosterols

Phytosterols were measured using LC-MS/MS according to the National Recommended Standard GB/T 39995-2021 of China, with minor modifications [23]. Phytosterols were separated on a C18 column (shim-pack GIST, 100 mm × 2.1 mm × 2 µm, Shimadzu, Kyoto, Japan) using a Shimadzu LCMS-8050 system equipped with an atmospheric pressure chemical ionization (APCI) source for the mass spectrometer (Shimadzu, Kyoto, Japan). Acetonitrile was used as phase A and water (0.1% formic acid) was used as phase B. The eluent gradient was programmed as follows: 80–100% A (0–2.5 min), 100% A (2.5–9 min), 100–80% A (9–9.1 min), and 80% A (9.1–15 min). The injection volume was 2 μL, with a 0.4 mL/min flow rate.

The APCI source of the mass spectrometer operated at a capillary voltage of 4.0 kV in the positive ion mode with the source temperature set at 300 °C and a desolvation gas flow of 10 L/min and desolvation temperature of 400 °C. Quantification of phytosterols was carried out using 6-Ketocholestanol as an internal standard.

#### 2.5.4. Total Phenolic Content (TPC)

Analysis of the total phenolic content (TPC) of *E. mollis* oil was determined as reported by Lee, et al. [12] using Folin-Ciocalteau method, with slight modifications. Briefly, the oil sample (0.2 g) was mixed with 2.5 mL methanol and 0.5 mL Folin-Ciocalteu reagent and shaken. Next, 1 mL Na_2_CO_3_ solution (75 g/L) was added to the mixture and the final volume was brought up to 10 mL with deionized water. The test was held in a dark place at room temperature for 2 h, then absorbance was read at 765 nm using a UV-2550 spectrophotometer (Shimadzu, Kyoto, Japan). Gallic acid was taken as the standard and the values were expressed as mg Gallic acid equivalents (GAE) per kg of sample oil (mg GAE/kg oil).

#### 2.5.5. Phenolic Compounds

For analysis of phenolic compounds, the oil sample (1.0 g) was dissolved in *n*-hexane (5 mL), then phenolic compounds were fractionated based on a modified method by Ribeiro, et al. [24] using weak cation exchange (WCX) solid-phase extraction cartridges. Ultra-high performance liquid chromatography with mass spectrometry (LCMS-8050, Shimadzu, Tokyo, Japan) was performed to identify and quantify individual phenolic compounds, and a C18 column (shim-pack GIST, 100 mm × 2.1 mm × 2 µm, Shimadzu, Kyoto, Japan) was used. Acetonitrile and formic acid (0.1%) were used as mobile phases A and B, with a flow rate of 0.3 mL/min. The gradient elution program comprised 10–95% A (0–3.5 min), 95% A (3.5–3.9 min), 95–10% A (3.9–4 min), 10% A (4–6 min).The capillary voltage was 4.0 kV, the desolvation gas flow 10 L/min, the source temperature 300 °C, and the desolvation temperature 400 °C. Electrospray ionization (ESI) (negative-ion mode) and multiple reaction monitoring (MRM) mode were used for quantitative analysis. Appropriate phenolic standards (4-hydroxybenzoic acid, caffeic acid, chlorogenic acid, cinnamic acid, gallic acid, vanillic acid, p-coumaric acid, sinapic acid, ferulic acid, ellagic acid, salicylic acid, vanillin, epicatechin, quercetin) were used for comparison and quantification; information about the quantification of the phenolic compounds (regression equations, R^2^, Linear range, quantitative ion, LOD, LOQ) is shown in Table 1 and Figure S1.

### 2.6. Antioxidant Activity

The antioxidant activity of *E. mollis* oil was evaluated based on DPPH radical scavenging activity according to the previous method reported by Kan, et al. [17]. *E. mollis* oil was dissolved in ethanol (concentrations of 2, 4, 8, 12, 16, 20, and 24 mg/mL). Two milliliters of sample solution were mixed with the same volume of DPPH (2 × 10^−4^ mol/L) ethanol solution. Then, the mixtures were shaken and kept in a dark place at room temperature for 30 min. The absorbance readings were recorded at 517 nm using a spectrophotometer (UV-2550, Shimadzu, Kyoto, Japan).

(1) $$DPPH\;radical\;scavenging\;activity\;\left(\%\right)=\left(\frac{A_{0}-A_{1}}{A_{0}}\right)\times 100$$

In this equation, *A*_0_ is the absorbance of the control group and *A*_1_ is the absorbance of the sample group.

### 2.7. Scanning Electron Micrographs (SEM)

Scanning electron micrographs (SEM) were used to reveal the microstructure of the raw materials according to the method of Liu, et al. [25] with slight modifications. The SEM images of surface morphological changes of unextracted and extracted seed powders were taken using a scanning electron microscope, FEI-Quanta 450 (Thermo Fisher Scientific, Waltham, MA, USA).

### 2.8. Thermal Stability

The thermal stability analysis of *E. mollis* oil was performed using a TGA 5500 (TA Instrument, DE, USA). A heating rate of 10 °C /min with a temperature in the range of 25–600 °C was used to carry out this analysis [26].

### 2.9. Statistical Analysis

All experiments were carried out in triplicate, and the results are presented as the mean value ± standard deviation. Statistical analysis was performed using Minitab 17 (Minitab Inc., Chicago, IL, USA) followed by Tukey’s test (*p* < 0.05).

## 3. Results and Discussion

### 3.1. Fatty Acid Profiles

Through chromatography–mass spectrometry twelve fatty acid components were identified, including seven saturated fatty acids (SFA), two monounsaturated fatty acids (MUFA) and three polyunsaturated fatty acids (PUFA) (Table 2). The primary fatty acids in *E. mollis* oil were linoleic acid (46.4~53.1%) and oleic acid (33.5~38.9%), which are considered to have high nutritional values thanks to their preventative effects against cardiovascular disease and cancer [27]. As seen in Table 2, the three oil products obtained from different extraction methods were rich in unsaturated fatty acids (90.9~93.96%) and the higher content of unsaturated fatty acids resulted from the UASEE process, which could cause a higher iodine value. Similar results were reported for the ultrasonic-enzymatic extraction of walnut oil [6]. Additionally, it was found that the most significant difference between three *E. mollis* oil products from different methods of extraction (UASEE, SE, and CP) was in the content rather than the components of fatty acids. The linoleic acid and polyunsaturated fatty acid content of the *E. mollis* oil obtained by the UASEE process (53.1% and 59.78%) was higher than that obtained by the SE (48.4% and 55.3%) and CP (46.4% and 53.62%) methods. Li, et al. [28] proposed that more polyunsaturated fatty acids in the dietary fats could prevent coronary heart and high blood pressure diseases. The reason for the higher content could be the relatively low extraction temperature and short extraction time of UASEE, which might help to prevent the oxidation and decomposition of unsaturated fatty acids [29].

### 3.2. Physicochemical Properties

Table 3 illustrates the physicochemical properties of oils obtained from different methods. The acid value was used to measure the acidity of the oil, and the peroxide value was used to measure the oxidation state of lipids [30,31]. Acid value and peroxide value determine the oxidation and rancidity of vegetable oil. As seen in Table 3, the UASEE oil product showed a significantly lower peroxide value (0.14 ± 0.01 g/100 g) and acid value (1.91 ± 0.02 mg/g) compared to SE oil (0.21 ± 0.03 g/100 g and 2.47 ± 0.04 mg/g), indicating that the oil obtained with the UASEE method exhibited a better quality with less rancidity [30]. Additionally, according to Ma, et al. [32], the lower acid value of UASEE oil could be the result of the neutralization of free fatty acids by the alkaline extraction environment during the extraction process, while the high temperature and prolonged extraction period during the SE process could be expected to accelerate the oxidative rancidity of oil [33].

The iodine value of the UASEE oil (162.96 ± 2.35 g/100 g) was slightly higher than that of the SE (151.35 ± 1.96 g/100 g) and CP (160.31 ± 1.87 g/100 g) oils (Table 3). Previous studies of the extraction of samara oil [34], walnut kernel oil [6], and *Sapindus mukorossi* seed kernel oil [35] all reported that the oil extracted by ultrasonic processes exhibited a higher iodine value and more unsaturated fatty acids than the SE products. These results are in good agreement with ours in the present study. There were no significant differences in the specific gravity, refractive index, and saponification values with the three methods, indicating that the purity and average molecular weight of fatty acids were similar [36]. Therefore, in terms of fatty acid profiles and physicochemical properties, UASEE served as the better alternative for the extraction of *E. mollis* oil.

### 3.3. Bioactive Compounds

#### 3.3.1. Tocopherols and Tocotrienols

Table 4 and Supplementary Figures S1 and S2illustrates the tocopherol and tocotrienol profiles and contents in oils obtained with the different methods. In this study, levels of total tocopherols and tocotrienols are expressed as the sum of α-tocopherol, γ-tocopherol, δ-tocopherol, α-tocotrienol, γ-tocotrienol, and δ-tocotrienol. The total tocopherol and tocotrienol contents in *E. mollis* oil ranged from 123.45 to 147.32 mg/100 g, similar to the results of Liang, et al. [37] (119.6–128.6 mg/100 g), although different from those of Wang, Duan, Fan and Li [8] (86.12–96.24 mg/100 g). This might be due to differences in geographical origin, cultivars, and analysis methods.

The contents of the total tocopherols and tocotrienols of *E. mollis* oil obtained using the UASEE method (147.32 ± 2.19 mg/100 g) were the highest of the three methods. It could be concluded that the total tocopherol and tocotrienol contents obtained depended on the extraction method. In the UASEE process, the interactivity between the tocopherols and tocotrienols and the protein was reduced by the enzymes, enhancing the release of tocopherols and tocotrienols into the oil [33,38,39]. The results showed that γ-tocopherol was the main component of *E. mollis* oil (111.75–134.50 mg/100 g), accounting for a proportion of more than 90%. The γ-tocopherol content showed a significant difference between UASEE (134.50 ± 2.62 mg/100 g) and SE (111.75 ± 1.86 mg/100 g). Mathur, et al. [40] reported that γ-tocopherol exerted a higher antioxidant capacity, anti-inflammatory, and cardioprotective effect than α-tocopherol. Therefore, UASEE seed oil is an ideal dietary source, with more antioxidants.

#### 3.3.2. Squalene

**Figure 1:**
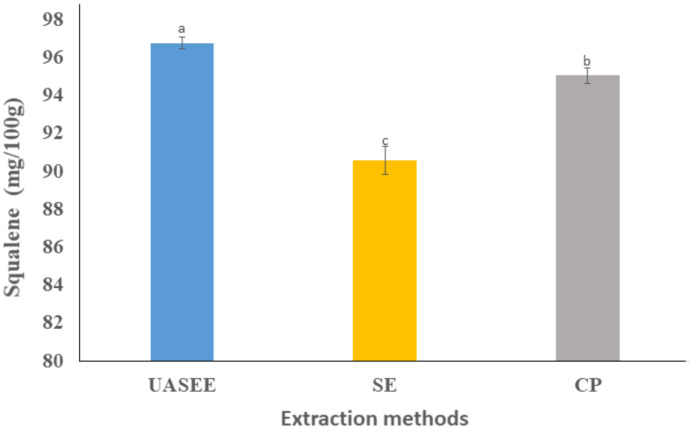
Squalene content of *E. mollis* oil obtained by different extraction methods. Different letters represent a significant difference at *p* < 0.05.

The squalene content of the oils is exhibited in Figure 1; the squalene content showed significant differences between the different extraction methods (*p* < 0.05). The highest squalene content was observed for UASEE oil (96.75 ± 0.31 mg/100 g), while the lowest was observed for SE oil (90.58 ± 0.74 mg/100 g). This illustrates that UASEE promotes the release of squalene. The chromatograms of squalene is showed in Supplementary Figures S3 and S4. The squalene content in this study was much higher compared with that of supercritical CO_2_ extraction (68.06 mg/100 g) [7]. This shows that different extraction methods have an impact on the content of squalene in seed oils.

#### 3.3.3. Phytosterols

As shown in Table 4, the contents of the individual phytosterols in *E. mollis* oil were significantly different due to the processing methods (*p* < 0.05). The major phytosterol in *E. mollis* oil Supplementary Figures S5–S7 was β-Sitosterol (from 139.03 ± 4.29 to 183.13 ± 4.62 mg/100 g), followed by Lupeol (from 35.78 ± 1.94 to 42.25 ± 1.37 mg/100 g), ergosterol (from 16.18 ± 0.83 to 20.64 ± 1.26 mg/100 g), stigmastanol (from 9.09 ± 0.17 to 9.36 ± 0.36 mg/100 g), and stigmasterol (from 6.53 ± 0.26 to 7.58 ± 0.24 mg/100 g). The total phytosterol content in *E. mollis* oil obtained by UASEE (261.78 ± 5.74 mg/100 g) was significantly (*p* < 0.05) higher compared with CP (207.66 ± 5.24 mg/100 g) and SE (247.82 ± 4.38 mg/100 g). This is consistent with the results reported by Konopka, et al. [41], who found that the ultrasound-solvent extraction oil and enzyme-extracted oil exhibited higher sterol contents due to the disruption of cell walls caused by enzymatic and ultrasonic treatment. Similarly, according to Fang, et al. [42], this combined treatment can reduce the interaction of phytosterols with the seed proteins, promoting their release into the oil.

#### 3.3.4. Total Phenolic Content and Profile Analysis

**Figure 2:**
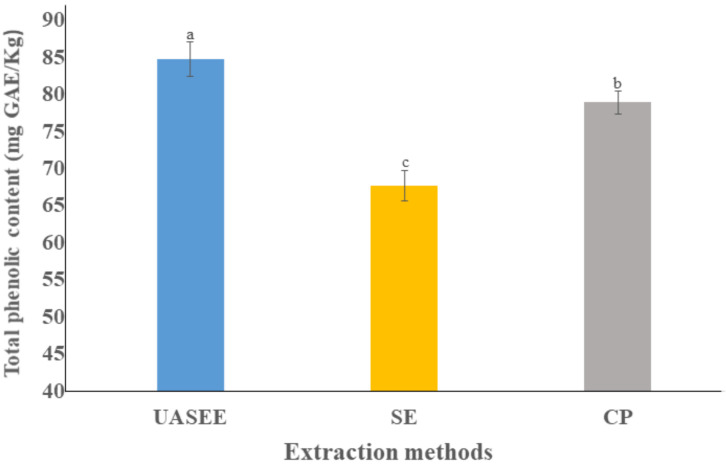
Total phenolic content of *E. mollis* oil obtained by different extraction methods. Different letters represent a significant difference at *p* < 0.05.

The total phenolic content (TPC) in *E.* *mollis* oil obtained by UASEE (84.76 ± 2.37 mg GAE/kg) was significantly higher compared to that obtained by SE (67.72 ± 2.04 mg GAE/Kg) and CP (78.90 ± 1.57 mg GAE/kg) (Figure 2). The observed difference may be due to the SE process requiring prolonged extraction times and high temperature, resulting in a reduction in TPC [43].

These results were further confirmed by the quantification of the phenolic compounds in three oils by HPLC-MS (Table 4 and Supplementary Figures S8–S10). In this study, four phenolic compounds were detected in *E. mollis* oil; ferulic acid was identified as the predominant phenolic compound (from 1.43 ± 0.10 to 2.53 ± 0.16 mg/kg), followed by p-coumaric acid, salicylic acid, and cinnamic acid. These phenolic compounds positively influence oil quality, oxidative stability, and shelf life, in addition to health benefits when consumed [24,44]. The phenolic components have several bioactive and pharmacological properties, including anti-inflammatory activity, and can help to prevent atherosclerosis and cancer [45].

As illustrated in Table 4, the UASEE oil presented significantly higher contents of all four phenolic compounds than oil from the other two extraction methods. One explanation for these observed differences in the contents of phenolic compounds may be that certain phenolic compounds might be more easily transferred to oil during the UASEE method. Similarly, Ghasemi and Dinani [6] demonstrated that ultrasonic-enzymatic extraction treatment using *n*-hexane was an effective way to extract walnut oil with higher levels of total phenolic content and iodine value than could be obtained with SE.

### 3.4. Antioxidant Activity

**Figure 3:**
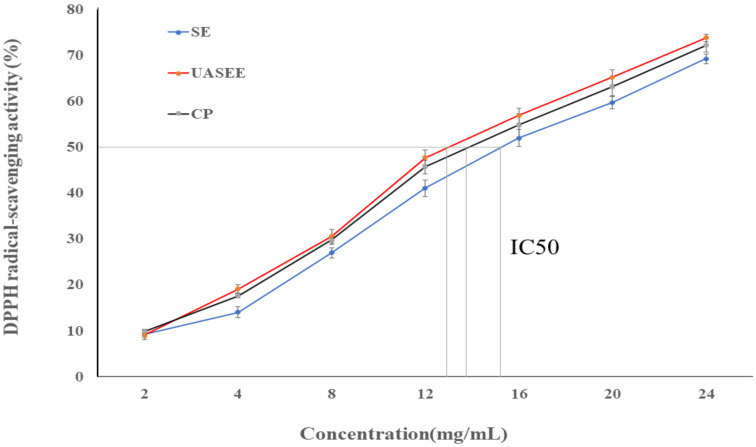
Antioxidant activity of *E.mollis* oil obtained by different methods.

The antioxidant activity of oil extracted by UASEE, SE, and CP was evaluated by DPPH radical scavenging assay. As shown in Figure 3, *E. mollis* oil’s antioxidant activity was dose-dependent within the experimental concentration range. With the increase in the concentration of *E. mollis* oil, the antioxidant capacity increased. The IC_50_ (half-maximal effective concentration) of the oil extracted by UASEE showed significant stronger antioxidant capacity (12.52 ± 0.28 mg/mL) compared with the SE (14.87 ± 0.48 mg/mL) and CP (13.29 ± 0.30 mg/mL) methods. This indicates that *E. mollis* oil had a strong scavenging activity on DPPH assay, and the highest antioxidant activity was observed for the UASEE method. The DPPH radical scavenging mechanism may occur through the *E. mollis* oil donating electrons or hydrogen atoms to DPPH [46]. The differences in *E. mollis* oil products in their bioactive compounds such as PUFA, phenolic compounds and unsaturated fatty acids affect their hydrogen-donating abilities [47]. In the present study, the excellent DPPH radical scavenging ability shown by *E. mollis* oil produced by UASEE process compared to the SE and CP processes may be the result of its higher content of bioactive compounds and its strong hydrogen-donating ability.

### 3.5. Microstructural Analysis

**Figure 4:**
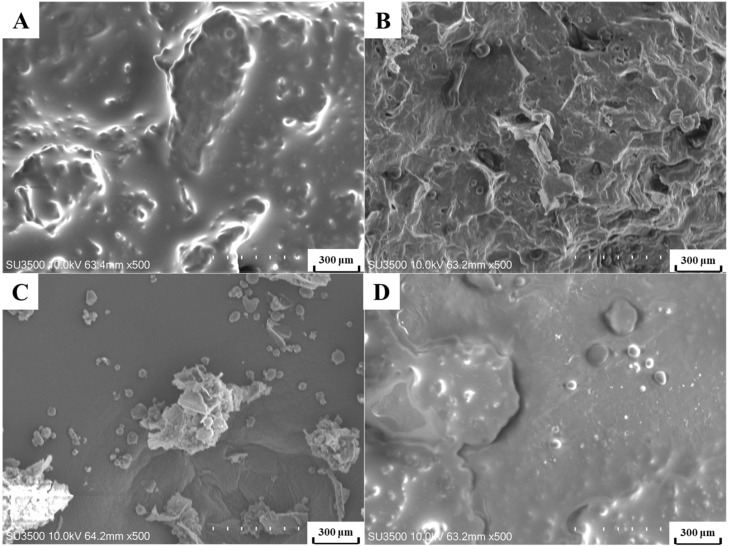
Scanning electron microscopy images of *E. mollis* powder: (**A**) Untreated, (**B**) UASEE residue, (**C**) SE residue, (**D**) CP residue.

Figure 4A shows scanning electron microscopy images of *E. mollis* seed before extraction, exhibiting a compact structure and no destruction of the surface of the cell walls. After SE, a loosened structure with thick porosity was found (Figure 4C). The CP-treated seed had a relatively loose external structure intact (Figure 4D). After extraction by UASEE (Figure 4B) the seed became porous, and most cells were curled, disorganized, and disrupted due to the loss of oil. Rosenthal, et al. [48] reported that the enzymatic treatment effectively destructed the cell walls and enhanced the oil released to the solvent. In addition, Zhang, et al. [49] and Jadhav, et al. [50] showed that the collapse of the cavities (bubbles) produced by ultrasonic pretreatment leads to a forceful shockwave and high-speed jet, which results in the creation of crevices, cracks, and micro-fractures. Therefore, the SEM images demonstrate that the enzymatic and ultrasonic treatment resulting in structural rupture of *E. mollis* seeds was helpful in releasing the oil from the raw materials.

### 3.6. Thermal Stability

**Figure 5:**
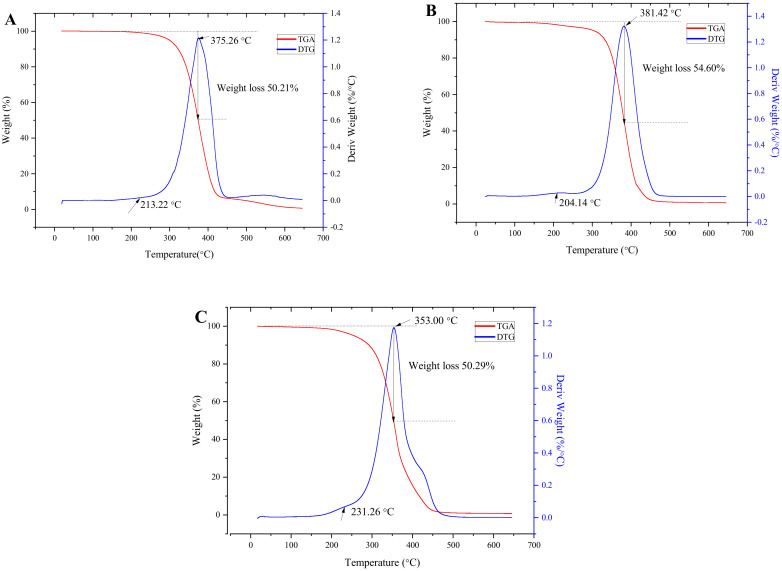
TGA and DTG curves of *E. mollis* oil obtained after three different processes: (**A**) UASEE, (**B**) SE, (**C**) CP.

Thermogravimetric analysis (TGA) and its corresponding derivative thermogravimetric analysis (DTG) can be used to analyze the thermal stability of *E. mollis* oil. Based on the TGA and DTG results, the thermal degradation of *E. mollis* oil was divided into two stages. The first degradation stage occurred at 152–251 °C, and was due to the decomposition of polyunsaturated fatty acids [51]. The second degradation stage occurred at 251–422 °C, and was the major weight loss stage. It could be attributed to changes in the chain length, degree of unsaturation, and branching of fatty acids [51,52]. Additionally, the DTG_MAX_ for the oils treated by UASEE, SE, and CP was observed at 375.26 °C, 381.32 °C, and 353.00 °C, respectively, while the respective weight loss of the oils was 50.21%, 54.60%, and 50.29% in this stage. The oil obtained by UASEE exhibited the lowest weight loss compared to CP oil and SE oil, suggesting its better thermal stability. As can be vividly seen in Figure 5, the degradation of *E. mollis* oil began at 152 °C; it can be assumed that the *E. mollis* oil would be thermally stable in industrial production with processing temperatures lower than 150 °C.

## 4. Conclusions

In this study, we compared the effects of UASEE and conventional processing methods (SE and CP) on the fatty acid content profiles, physicochemical properties, bioactive compounds, antioxidant activity, and thermal behavior of *E. mollis* oil.

All the results illustrate that UASEE has an important effect on oil quality. The UASEE oil exhibited more advantages in functionality, including more unsaturated fatty acids, tocopherols and tocotrienols, squalene, phytosterols, and phenolic contents. The oil obtained by UASEE showed a relatively lower weight loss than the other two oils, suggesting its better thermal stability. Scanning electron microscopy showed damage to the cell walls and release of substances from cells with UASEE. Thus, through evaluation of its physicochemical properties, functional compounds, antioxidant activity, and thermal stability, the *E. mollis* oil obtained with the UASEE process has potential for application as a natural antioxidant or functional regredient applied in the future food process industry, thanks to its excellent DPPH radical scavenging activity and the advantages of abundant unsaturated fatty acids and bioactive compounds. The present study provides ideas for the further development of *E. mollis* oil-related functional foods.

## Figures and Tables

**Table 1:** The quantification information of phenolics in *E. mollis* oil.

No.	Compounds	Regression Equation	R^2^	Linear Range (μg/L)	*m*/*z*	RT (min)	LOD (mg/kg)	LOQ (mg/kg)
1	chlorogenic acid	Y = 13025.5X + 9211.17	0.9999	2–500	353.1/191.1	1.597	1.5	5.0
2	cinnamic acid	Y = 1833.16X + 5305.62	0.9987	2–500	149.0/131.0	2.263	1.5	5.0
3	gallic acid	Y = 422.457X + 142.217	0.9998	2–200	169.0/79.1	2.360	0.15	0.5
4	vanillic acid	Y = 150.973X + 1120.40	0.9973	2–200	167.1/108.0	2.588	0.3	1.0
5	p-coumaric acid	Y = 9272.58X + 12986.6	0.9999	2–200	163.0/119.1	2.555	0.3	1.0
6	sinapic acid	Y = 1819.15X − 4545.19	0.9990	2–500	223.1/164.1	2.655	1.5	5.0
7	ferulic acid	Y = 2826.58X − 229.223	0.9999	2–200	193.1/134.1	2.871	0.3	1.0
8	ellagic acid	Y = 175.979X − 1233.08	0.9990	2–200	301.2/229.0	2.913	0.3	1.0
9	salicylic acid	Y = 18774.9X + 97389.3	0.9999	2–200	137.1/93.1	2.943	0.3	1.0
10	4-hydroxybenzoic acid	Y = 8625.58X + 27775.2	0.9997	2–200	137.1/93.1	2.973	0.15	0.5
11	vanillin	Y = 11935.6X + 31234.6	0.9999	2–200	153.2/93.2	3.305	0.3	1.0
12	epicatechin	Y = 1594.97X − 4848.19	0.9983	2–800	305.1/125.0	3.307	5.0	20.0
13	quercetin	Y = 4288.29X − 7960.59	0.9996	2–200	301.0/151.0	3.309	0.15	0.5
14	caffeic acid	Y = 9052.98X + 16558.7	0.9999	2–200	179.1/135.1	3.519	0.3	1.0

**Table 2:** Fatty acid profiles of *E.mollis* oil extracted by three methods.

Fatty Acid Profiles (%)	Extraction Methods		
	UASEE	SE	CP
Myristic (14:0)	0.02 ± 0.00 ^a^	0.02 ± 0.00 ^a^	0.02 ± 0.00 ^a^
Pentadecanoic (15:0)	0.02 ± 0.00 ^a^	0.02 ± 0.00 ^a^	0.02 ± 0.00 ^a^
Palmitic acid (16:0)	3.42 ± 0.13 ^c^	5.72 ± 0.26 ^a^	4.03 ± 0.13 ^b^
Margaric (17:0)	0.05 ± 0.00 ^a^	0.05 ± 0.00 ^a^	0.05 ± 0.00 ^a^
Stearic acid (18:0)	2.15 ± 0.15 ^b^	2.91 ± 0.14 ^a^	2.42 ± 0.20 ^b^
Arachidic acid (20:0)	0.32 ± 0.01 ^a^	0.35 ± 0.02 ^a^	0.21 ± 0.01 ^b^
Behenic (22:0)	0.06 ± 0.00 ^a^	0.06 ± 0.00 ^a^	0.06 ± 0.00 ^a^
Saturated fatty acids (SFA)	6.04 ± 0.29 ^c^	9.13 ± 0.41 ^a^	6.81 ± 0.15 ^b^
Oleic acid (18:1)	33.5 ± 0.21 ^c^	34.9 ± 0.20 ^b^	38. 9 ± 0.15 ^a^
Eicosenoic acid (20:1)	0.64 ± 0.01 ^b^	0.70 ± 0.01 ^a^	0.64 ± 0.03 ^b^
Monounsaturated fatty acids (MUFA)	34.18 ± 0.21 ^c^	35.60 ± 0.21 ^b^	39.57 ± 0.18 ^a^
Eicosadienoic (20:2)	0.10 ± 0.00 ^a^	0.10 ± 0.01 ^a^	0.10 ± 0.01 ^a^
Linoleic acid (18:2)	53.1 ± 0.35 ^a^	48.4 ± 0.67 ^b^	46.4 ± 0.38 ^c^
α-linolenic acid (18:3)	6.55 ± 0.12 ^b^	6.84 ± 0.27 ^b^	7.09 ± 0.13 ^a^
Polyunsaturated fatty acids (PUFA)	59.78 ± 0.44 ^a^	55.30 ± 0.60 ^b^	53.62 ± 0.24 ^b^
Unsaturated fatty acids (UFA)	93.96 ± 0.28 ^a^	90.9 ± 0.35 ^b^	93.19 ± 0.19 ^b^

Values are means ± SD (*n* = 3). Different letters within a row represent significant difference at *p* < 0.05.

**Table 3:** Oil yield and physicochemical properties of *E.mollis* oil extracted by three methods.

Physicochemical Properties	Extraction Methods		
	UASEE	SE	CP
Oil yield (%)	43.35 ± 0.26 ^a^	43.02 ± 0.77 ^a^	22.05 ± 0.12 ^b^
Specific gravity (20 °C/g/mL)	0.9150 ± 0.00 ^a^	0.9149 ± 0.00 ^a^	0.9149 ± 0.00 ^a^
Refractive index (20 °C)	1.474 ± 0.00 ^a^	1.474 ± 0.00 ^a^	1.474 ± 0.00 ^a^
Acid value (mg/g)	1.91 ± 0.02 ^a^	2.47 ± 0.04 ^b^	1.87 ± 0.03 ^a^
Peroxide value (g/100 g)	0.14 ± 0.01 ^a^	0.21 ± 0.03 ^b^	0.12 ± 0.02 ^a^
Iodine value (g/100 g)	162.96 ± 2.35 ^a^	151.35 ± 1.96 ^b^	160.31 ± 1.87 ^a^
Saponification value (mg/g)	174.47 ± 2.60 ^a^	174.48 ± 2.13 ^a^	176.77 ± 1.49 ^a^

Values are means ± SD (*n* = 3). Different letters within a row represent a significant difference at *p* < 0.05.

**Table 4:** Bioactive compounds and antioxidant activity of *E.mollis* oil extracted by three methods.

Bioactive Compounds	Extraction Methods		
	UASEE	SE	CP
α-tocopherol (mg/100 g)	3.58 ± 0.26 ^a^	2.70 ± 0.15 ^b^	3.34 ± 0.23 ^a^
β-tocopherol (mg/100 g)	ND	ND	ND
γ-tocopherol (mg/100 g)	134.50 ± 2.62 ^a^	111.75 ± 1.86 ^b^	132.08 ± 2.39 ^a^
δ-tocopherol (mg/100 g)	1.67 ± 0.05 ^a^	1.71 ± 0.02 ^b^	1.69 ± 0.04 ^a^
α-tocotrienol (mg/100 g)	1.13 ± 0.11 ^b^	1.53± 0.14 ^a^	1.52 ± 0.06 ^a^
β-tocotrienol (mg/100 g)	ND	ND	ND
γ-tocotrienol (mg/100 g)	5.72 ± 0.21 ^a^	4.97 ± 0.16 ^b^	5.42 ± 0.12 ^a^
δ-tocotrienol (mg/100 g)	0.71 ± 0.07 ^a^	0.79 ± 0.05 ^a^	0.80 ± 0.04 ^a^
Total tocopherol and tocotrienol (mg/100 g)	147.32 ± 2.19 ^a^	123.45 ± 1.74 ^b^	144.84 ± 2.24 ^a^
β-Sitosterol (mg/100 g)	183.13 ± 4.62 ^a^	170.23 ± 5.18 ^b^	139.03 ± 4.29 ^c^
Stigmasterol (mg/100 g)	6.53 ± 0.26 ^b^	6.72 ± 0.42 ^b^	7.58 ± 0.24 ^a^
Ergosterol (mg/100 g)	20.64 ± 1.26 ^a^	20.35 ± 1.19 ^a^	16.18 ± 0.83 ^b^
Lupeol (mg/100 g)	42.25 ± 1.37 ^a^	41.17 ± 0.68 ^a^	35.78 ± 1.94 ^b^
Stigmastanol (mg/100 g)	9.24 ± 0.20 ^a^	9.36 ± 0.36 ^a^	9.09 ± 0.17 ^a^
Total Phytosterol (mg/100 g)	261.78 ± 5.74 ^a^	247.82 ± 4.38 ^b^	207.66 ± 5.24 ^c^
Salicylic acid (mg/kg)	0.97 ± 0.03 ^a^	0.79 ± 0.01 ^c^	0.86 ± 0.03 ^b^
Ferulic acid (mg/kg)	2.53 ± 0.16 ^a^	1.43 ± 0.10 ^c^	2.09 ± 0.12 ^b^
Cinnamic acid (mg/kg)	0.66 ± 0.03 ^a^	0.63 ± 0.01 ^a^	0.65 ± 0.02 ^a^
p-Coumaric acid (mg/kg)	1.27 ± 0.06 ^a^	0.91 ± 0.07 ^c^	1.09 ± 0.03 ^b^
DPPH (IC 50) (mg/mL)	12.52 ± 0.28 ^a^	14.87 ± 0.48 ^b^	13.29 ± 0.30 ^a^

ND: not detected. Values are means ± SD (*n* = 3). Different letters within a row represent a significant difference at *p* < 0.05.

## Data Availability

Data available on request from the authors.

## Supplementary Materials

The following are available online at https://www.mdpi.com/article/10.3390/foods11030359/s1, Figure S1: Chromatograms of tocopherols and tocotrienols standard solution. Figure S2: Chroma-tograms of tocopherols and tocotrienols sample solution of UASEE oil. Figure S3: Quantitative ion chromatograms of squalene standard solution. Figure S4: Quantitative ion chromatograms of squalene sample solution of UASEE oil. Figure S5: Total ion chromatograms (TIC) of phytosterols standard solution. Figure S6: Quantitative ion chromatograms of phytosterols standard solution. Figure S7: Quantitative ion chromatograms of phytosterols sample solution of UASEE oil. Figure S8: Total ion chromatograms (TIC) of phenolic compounds standard solution. Figure S9: Quanti-tative ion chromatograms of phenolic compounds standard solution. Figure S10: Quantitative ion chromatograms of phenolic compounds sample solution of UASEE oil.
