# Supplementary material for: Effect of Ultrasound-Assisted Solvent Enzymatic Extraction on Fatty Acid Profiles, Physicochemical Properties, Bioactive Compounds, and Antioxidant Activity of Elaeagnus mollis Oil
Source: Foods. 2022 Jan 26;11(3):359. doi: 10.3390/foods11030359 (PMC8834463; doi:10.3390/foods11030359)
Supplement: Supplementary file 1 [file foods-11-00359-s001.zip › foods-1569231 suppl final/foods-1569231 suppl final.pdf]

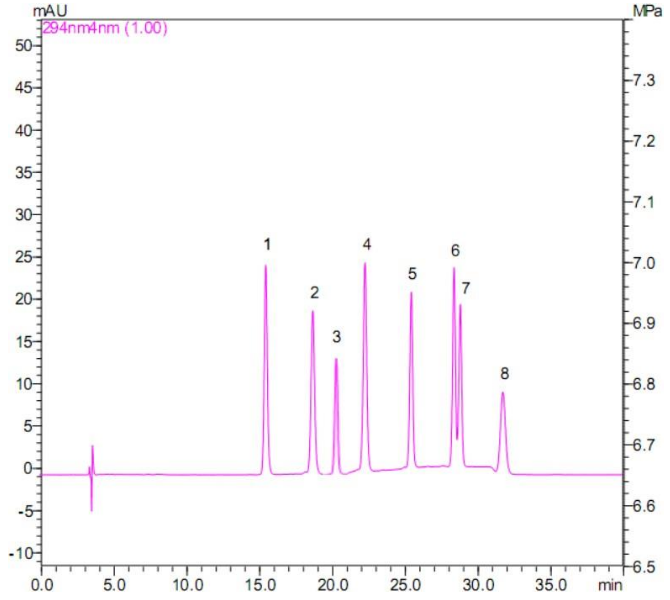

Peak number, 1:δ-tocotrienol, 2:γ-tocotrienol, 3:β-tocotrienol, 4:α-tocotrienol, 5:δ-tocopherol, 6:γ-tocopherol, 7:β-tocopherol, 8:α-tocopherol

Figure S1. Chromatograms of tocopherols and tocotrienols standard solution

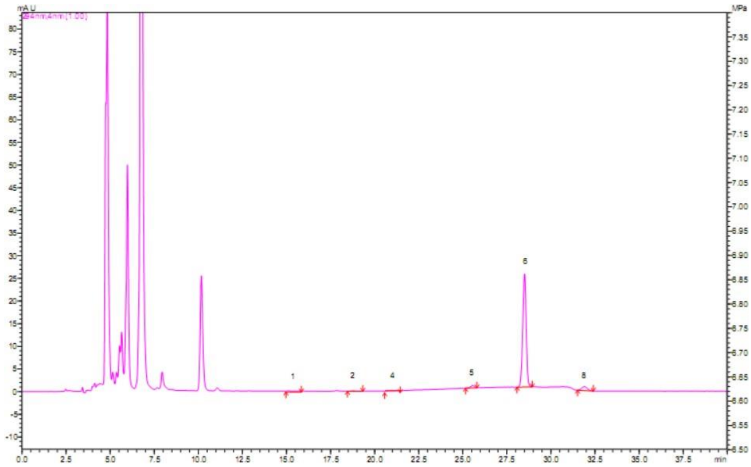

Peak numbering refers to peaks in Figure S1.

Figure S2. Chromatograms of tocopherols and tocotrienols sample solution of UASEE oil.

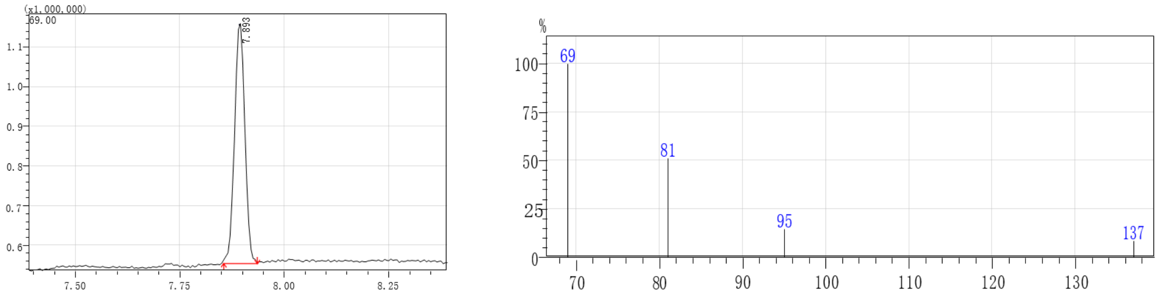

Figure S3. Quantitative ion chromatograms of squalene standard solution.

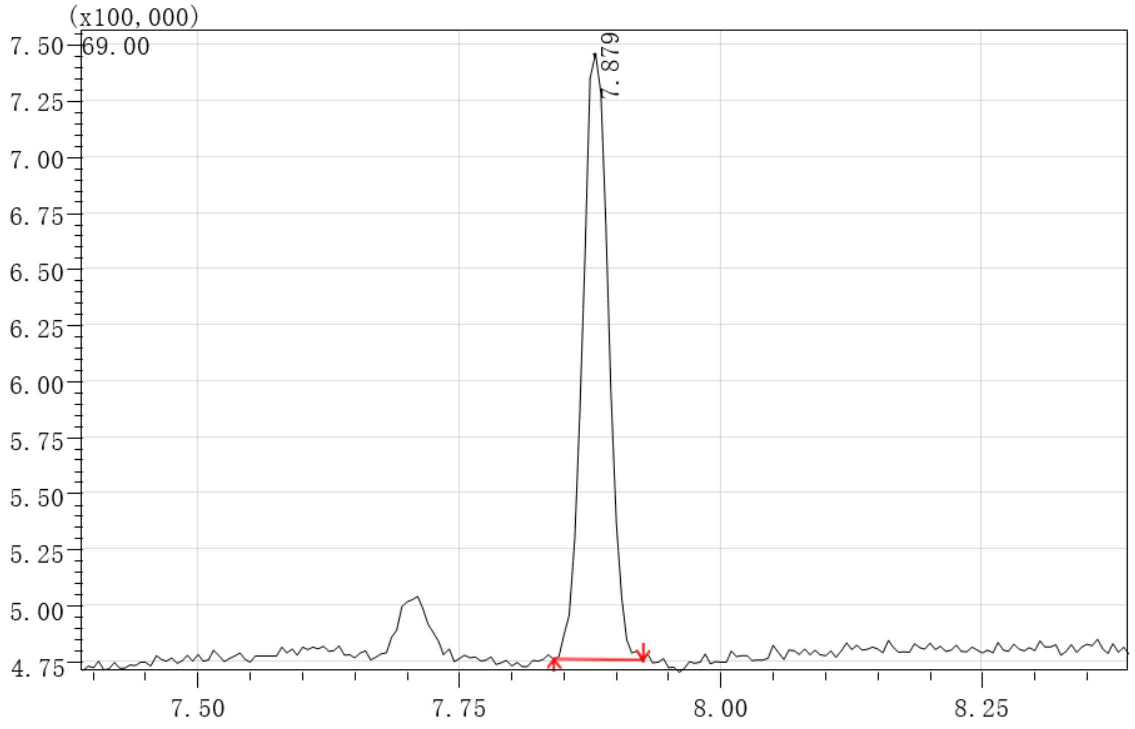

Figure S4. Quantitative ion chromatograms of squalene sample solution of UASEE oil.

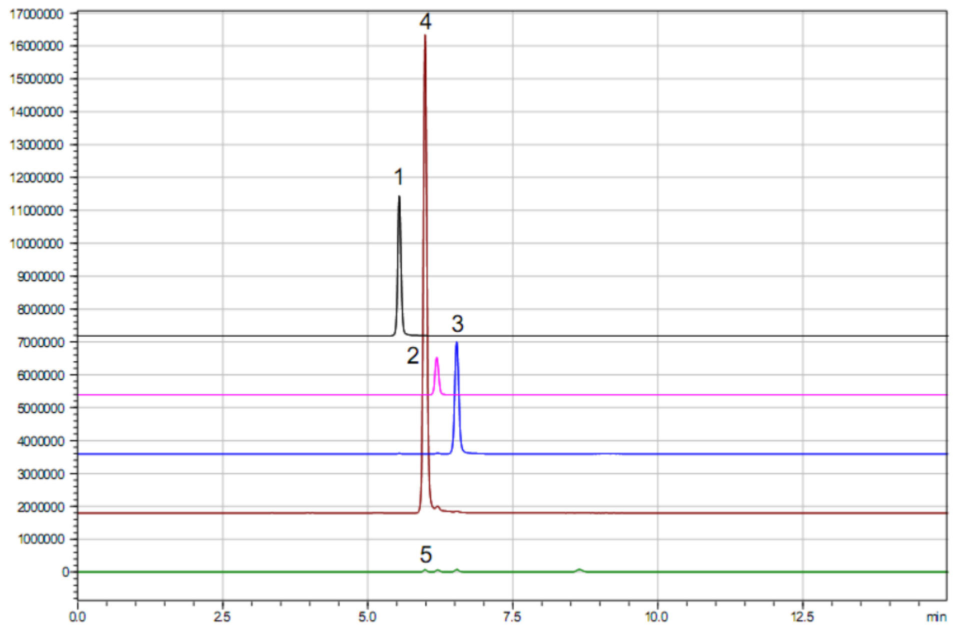

Peak number, 1:Ergosterol, 2:Stigmastanol, 3:β-Sitosterol, 4:Lupeol, 5:Stigmasterol

Figure S5. Total ion chromatograms (TIC) of phytosterols standard solution

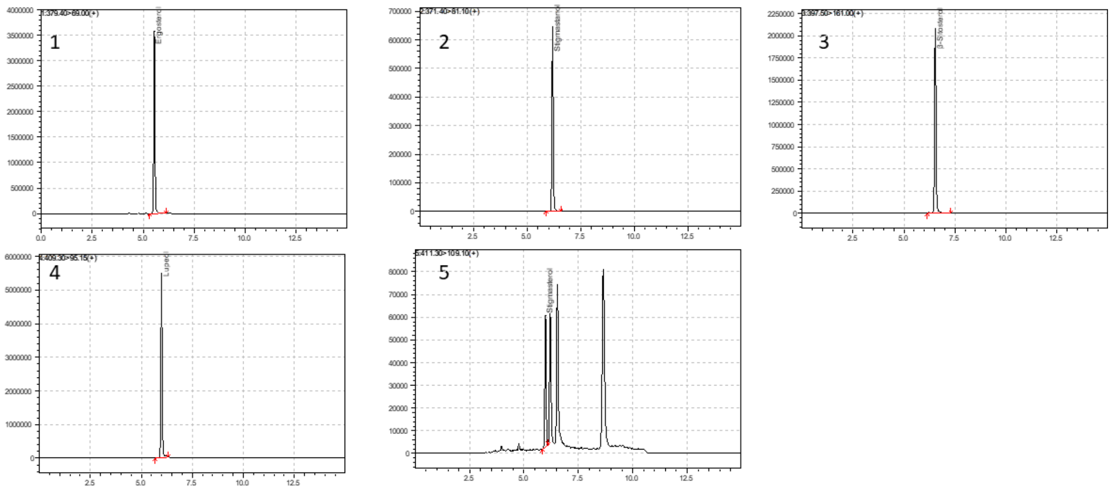

Peak numbering refers to peaks in Figure S5.

Figure S6. Quantitative ion chromatograms of phytosterols standard solution

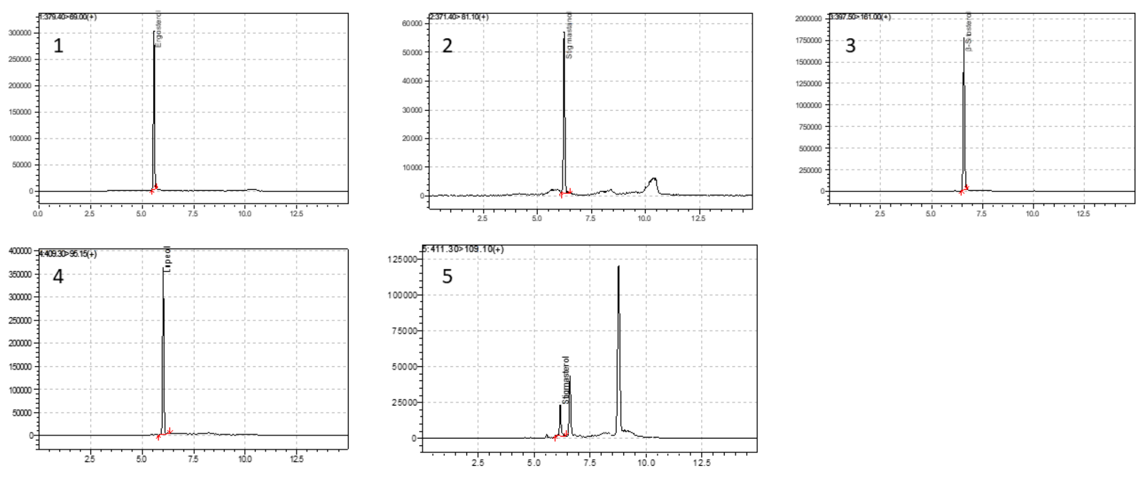

Peak numbering refers to peaks in Figure S5.

Figure S7. Quantitative ion chromatograms of phytosterols sample solution of UASEE oil.

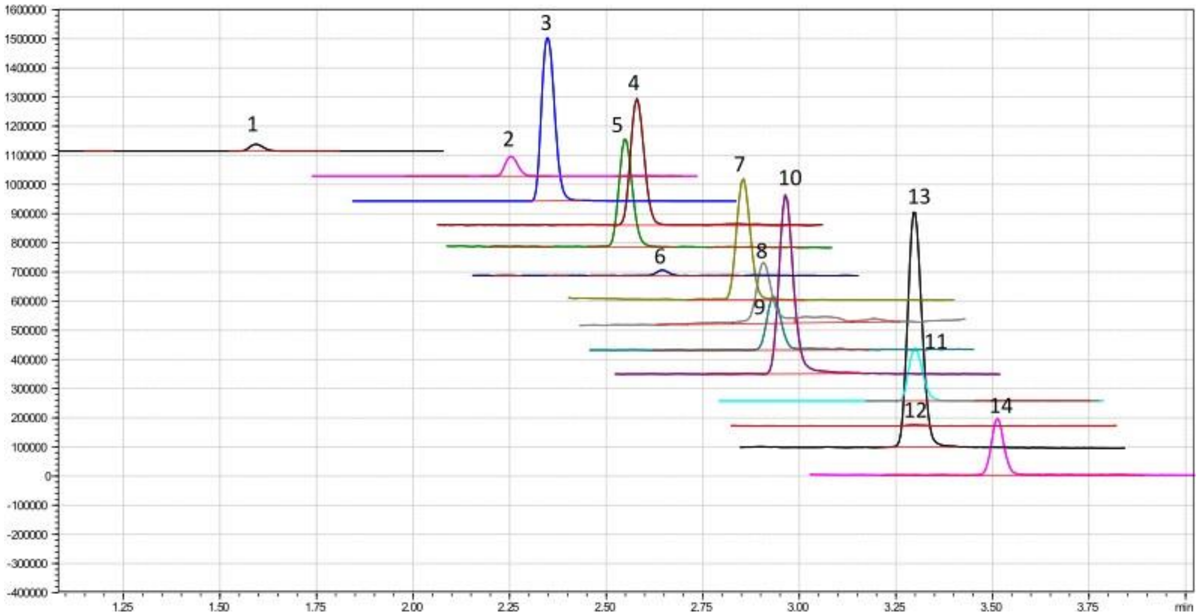

Peak number,1: chlorogenic acid, 2: cinnamic acid, 3:gallic acid, 4:vanillic acid, 5:p-coumaric acid, 6:sinapic acid, 7:ferulic acid, 8:ellagic acid, 9:salicylic acid,10:4- hydroxybenzoic acid, 11:vanillin, 12:epicatechin, 13:quercetin, 14:caffeic acid

Figure S8.Total ion chromatograms (TIC) of phenolic compounds standard solution

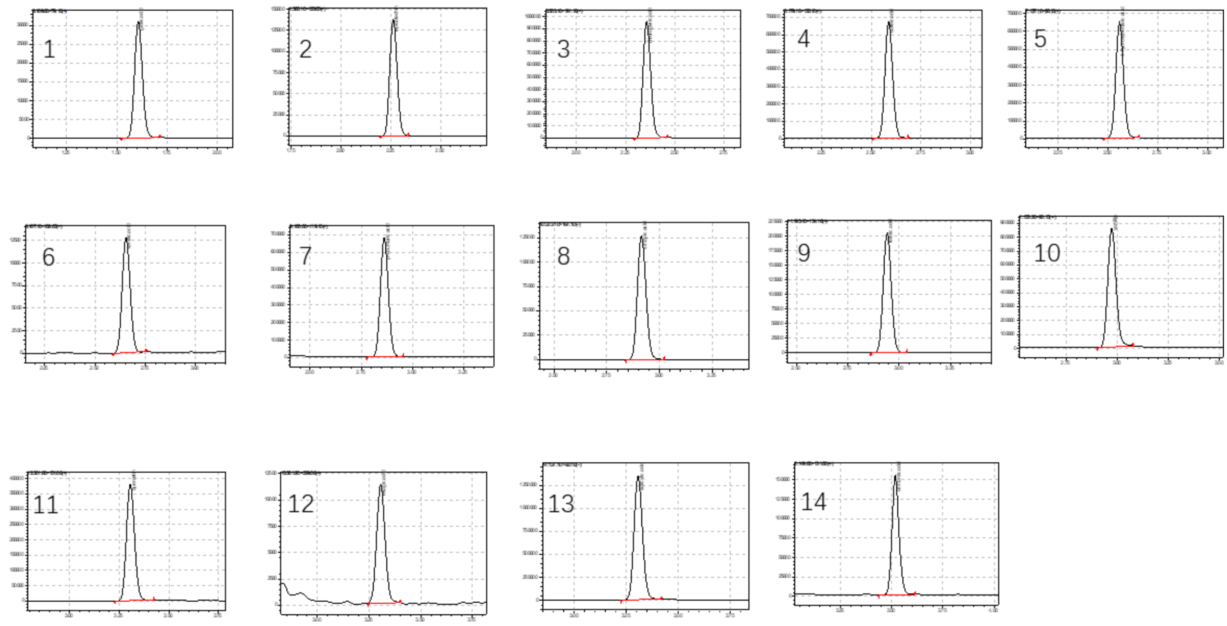

Peak numbering refers to peaks in Figure S8.

Figure S9. Quantitative ion chromatograms of phenolic compounds standard solution

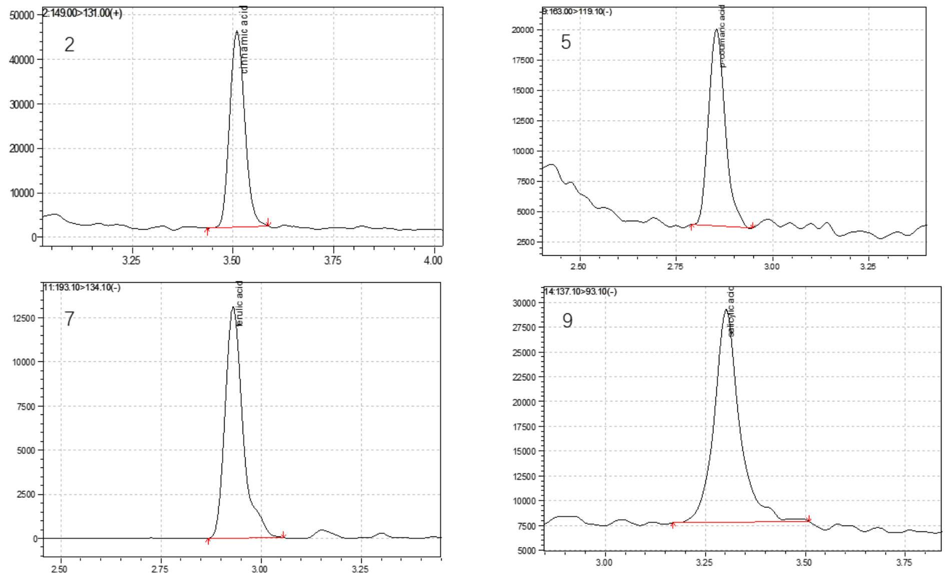

Peak numbering refers to peaks in Figure S8.

Figure S10. Quantitative ion chromatograms of phenolic compounds sample solution of UASEE oil.
